# Adverse Childhood Experiences During the COVID-19 Pandemic and Associations with Poor Mental Health and Suicidal Behaviors Among High School Students — Adolescent Behaviors and Experiences Survey, United States, January–June 2021

**DOI:** 10.15585/mmwr.mm7141a2

**Published:** 2022-10-14

**Authors:** Kayla N. Anderson, Elizabeth A. Swedo, Eva Trinh, Colleen M. Ray, Kathleen H. Krause, Jorge V. Verlenden, Heather B. Clayton, Andrés Villaveces, Greta M. Massetti, Phyllis Holditch Niolon

**Affiliations:** ^1^Division of Violence Prevention, National Center for Injury Prevention and Control, CDC; ^2^Division of Injury Prevention, National Center for Injury Prevention and Control, CDC; ^3^Division of Adolescent and School Health, National Center for HIV, Viral Hepatitis, STD, and TB Prevention, CDC.

Social and educational disruptions during the COVID-19 pandemic have exacerbated concerns about adolescents’ mental health and suicidal behavior. Data from the 2021 Adolescent Behaviors and Experiences Survey (ABES) indicate that 37.1% of U.S. high school students reported poor mental health during the COVID-19 pandemic, with 19.9% considering and 9.0% attempting suicide in the preceding year (*1*). Adverse childhood experiences (ACEs)* are associated with poor mental health and suicidal behaviors (*2*,*3*), and high prevalence of some ACEs have been documented during the pandemic (*4*). ACEs are preventable, potentially traumatic events that occur in childhood (ages 0–17 years) such as neglect, experiencing or witnessing violence, or having a family member attempt or die by suicide. Also included are aspects of a child’s environment that can undermine their sense of safety, stability, and bonding. Associations between ACEs occurring during the pandemic and mental health or suicidal behaviors among U.S. high school students were examined using ABES data. Experience of one to two ACEs was associated with poorer mental health and increased suicidal behaviors, and these deleterious outcomes increased with additional ACE exposure. After adjusting for demographic characteristics, adolescents who reported four or more ACEs during the pandemic had a prevalence of poor current mental health four times as high as, and a prevalence of past-year suicide attempts 25 times as high as, those without ACEs during the pandemic. Experience of specific ACE types (e.g., emotional abuse) was associated with higher prevalences of poor mental health and suicidal behaviors. Prevention and intervention strategies (*5*), including early identification and trauma-informed mental health service and support provision, for ACEs and their acute and long-term impacts could help address the U.S. child and adolescent mental health and suicide crisis.^†^

The voluntary, probability-based online ABES used stratified, three-stage cluster sampling to obtain nationally representative data from U.S. public and private high school students in grades 9–12 during January–June 2021^§^ (*1*,*4*). Prevalence estimates for some ACEs, mental health, and suicidal behaviors have been reported previously (*1*,*4*). Students self-reported experiences of some adversities during the COVID-19 pandemic (i.e., emotional abuse, physical abuse, parent or caregiver job loss, and food insecurity) or during the past 12 months^¶^ (i.e., sexual violence by any perpetrator, physical teen dating violence, and electronic bullying), as well as their mental health (i.e., current poor mental health, poor mental health during the pandemic, and persistent feelings of sadness or hopelessness during the past year) and suicidal behaviors (i.e., seriously considering suicide, making a suicide plan, or attempting suicide during the past year) (Supplementary Box, https://stacks.cdc.gov/view/cdc/121661).

The analysis was restricted to 4,390 high school students aged <18 years with complete data on analytic variables, to align with the ACEs focus. ACEs were examined by type (e.g., emotional abuse), category (e.g., exposure to either of the two abuse-related ACEs), and cumulative number of ACEs (zero, one to two, three, and four or more). Weighted pairwise prevalence estimates and 95% CIs for reported ACE exposure by poor mental health or suicidal behaviors were calculated. Adjusted prevalence ratios (aPRs) and 95% CIs were calculated using Poisson regression with robust SEs to examine associations between reported ACE exposure during the pandemic and mental health or suicidal behaviors, with and without inclusion of other ACEs. aPRs were adjusted for sex, grade in school, race and ethnicity, sexual identity, and method of school instruction at recruitment into ABES; 95% CIs that excluded 1.0 were considered statistically significant. The ABES study protocol was reviewed and approved by the institutional review boards at CDC and ICF International, CDC’s survey contractor.** Analyses were conducted using SAS statistical software (version 9.4, SAS Institute), accounting for complex survey design.

Nearly three quarters (73.1%) of high school students aged <18 years reported at least one ACE during the COVID-19 pandemic (53.2%, 12.0%, and 7.8% reported one to two, three, and four or more ACEs, respectively (mean = 1.47; SE = 0.04). Compared with adolescents without ACEs, adolescents who reported one to two ACEs during the pandemic had higher prevalences of poor mental health and suicidal behaviors (aPR range = 1.97–2.39 and 3.29–5.92, respectively) (Table 1). A dose-response relationship between accumulating exposure to ACEs during the pandemic and poor mental health and suicidal behaviors was observed (Figure). Compared with adolescents without ACEs, adolescents with four or more ACEs during the pandemic had a prevalence of poor mental health three to four times as high (aPR range = 3.04–4.06) as well as substantially higher prevalences of past-year suicidal behaviors (seriously considering suicide: 57.4% versus 5.3%, aPR = 7.06, 95% CI = 5.02–9.93; making a suicide plan: 48.6% versus 3.7%, aPR = 8.27, 95% CI = 5.09–13.42; attempted suicide: 38.7% versus 0.9%, aPR = 25.06, 95% CI = 11.35–55.30), after adjusting for demographic characteristics (Table 1).

**TABLE 1:** Associations between number of adverse childhood experiences during the COVID–19 pandemic and poor mental health and suicidal behaviors among high school students (N = 4,390) — Adolescent Behaviors and Experiences Survey, United States, 2021

Indicator	aPR* (95% CI)			
	No ACEs (n = 1,167)	One or two ACEs (n = 2,358)	Three ACEs (n = 512)	Four or more ACEs (n = 353)
**Poor mental health**				
Current poor mental health (past 30 days)	Ref	2.39 (1.96–2.92)	3.22 (2.59–3.99)	4.06 (3.31–4.99)
Poor mental health during the COVID–19 pandemic	Ref	2.13 (1.84–2.48)	2.72 (2.29–3.23)	3.18 (2.71–3.74)
Persistent feelings of sadness or hopelessness during the past year	Ref	1.97 (1.72–2.26)	2.80 (2.41–3.24)	3.04 (2.63–3.52)
**Suicidal behaviors**				
Seriously considered suicide during the past year	Ref	3.29 (2.31–4.69)	5.13 (3.54–7.45)	7.06 (5.02–9.93)
Made a suicide plan during the past year	Ref	3.29 (2.02–5.35)	5.85 (3.48–9.83)	8.27 (5.09–13.42)
Attempted suicide during the past year	Ref	5.92 (2.71–12.94)	15.85 (7.35–34.19)	25.06 (11.35–55.30)

**Abbreviations:** ACEs = adverse childhood experiences; aPR = adjusted prevalence ratio; Ref = referent group.

* Adjusted for sex, race and ethnicity, grade in school, sexual identity, and method of school instruction.

**FIGURE:**
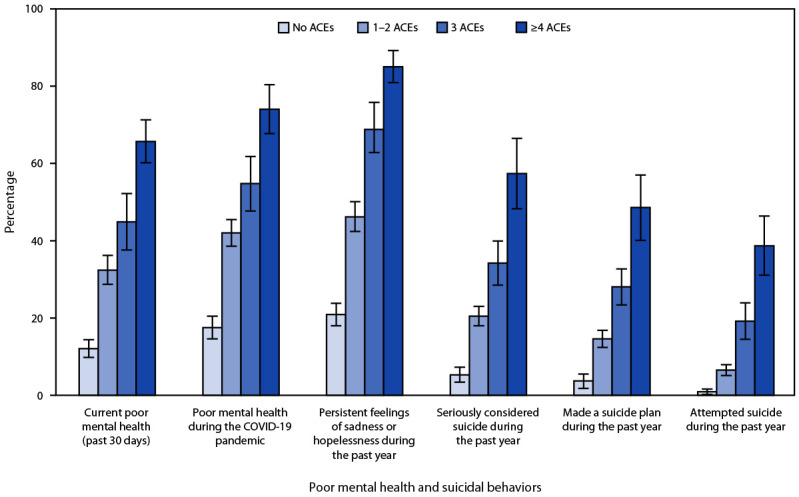
Number of adverse childhood experiences during the COVID-19 pandemic and crude prevalences of poor mental health and suicidal behaviors* among high school students (N = 4,390) — Adolescent Behaviors and Experiences Survey, United States, 2021 **Abbreviation:** ACEs = adverse childhood experiences. * 95% CIs indicated by error bars.

After adjusting for demographic characteristics, experience of each ACE type was associated with a higher prevalence of current poor mental health (aPR range = 1.26–2.22), poor mental health during the COVID-19 pandemic (aPR range = 1.28–1.87), and past-year persistent feelings of sadness or hopelessness (aPR range = 1.21–1.94) (Table 2). The prevalence of poor mental health among adolescents who reported specific types of ACEs was high. For example, 82.7% and 82.0% who experienced past-year sexual violence or physical teen dating violence, respectively, felt persistently sad or hopeless. Likewise, exposure to each ACE type was associated with a higher prevalence of seriously considering suicide (aPR range = 1.23–3.13), making a suicide plan (aPR range = 1.74–3.62 [except for parent or caregiver job loss]), and attempting suicide in the past year (aPR range = 1.45–5.42). Suicidal behavior prevalence by each ACE type was high; for example, 33.0% of adolescents who experienced any sexual violence during the past year reported attempting suicide. After adjusting for all other assessed ACEs and demographic characteristics, emotional abuse was most strongly associated with poor mental health (aPRs for poor current mental health, poor mental health during the pandemic, and past-year persistent sadness or hopelessness were 1.96, 1.68, and 1.73, respectively) and past-year suicidal behaviors (aPRs for having seriously considered suicide, made a suicide plan, and attempted suicide were 2.61, 2.89, and 3.62, respectively). Experience of any abuse-related ACEs during the pandemic was more strongly associated with mental health and suicidal behaviors than was experience of other forms of violence or family economic pressures (Supplementary Table, https://stacks.cdc.gov/view/cdc/121662).

**TABLE 2:** Associations between types of adverse childhood experiences during the COVID–19 pandemic and poor mental health and suicidal behaviors among high school students (N = 4,390) — Adolescent Behaviors and Experiences Survey, United States, 2021

Indicator		Physical abuse during the COVID-19 pandemic		Emotional abuse during the COVID-19 pandemic		Sexual violence during the past year		Physical teen dating violence during the past year		Electronic bullying during the past year		Parent lost job during the COVID-19 pandemic		Food insecurity during the COVID-19 pandemic	
		Yes	No	Yes	No	Yes	No	Yes	No	Yes	No	Yes	No	Yes	No
		(n = 519)	(n = 3,871)	(n = 2,547)	(n = 1,843)	(n = 474)	(n = 3,916)	(n = 140)	(n = 4,250)	(n = 599)	(n = 3,791)	(n = 1,226)	(n = 3,164)	(n = 959)	(n = 3,431)
**Poor mental health**															
Current poor mental health (past 30 days)	% Yes, 95% CI	51.8 (45.2–58.4)	28.5 (25.6–31.4)	42.3 (38.5–46.1)	16.1 (13.6–18.6)	58.3 (53.1–63.6)	28.1 (25.1–31.1)	56.3 (44.3–68.3)	30.4 (27.3–33.4)	51.7 (45.9–57.5)	27.9 (25.1–30.7)	37.9 (33.1–42.8)	28.2 (25.5–30.9)	43.3 (39.6–47.0)	27.6 (24.4–30.8)
	aPR,* 95% CI	1.66 (1.47–1.88)	—	2.22 (1.93–2.56)	—	1.53 (1.34–1.76)	—	1.52 (1.25–1.86)	—	1.55 (1.37–1.74)	—	1.26 (1.13–1.42)	—	1.51 (1.34–1.69)	—
	aPR,^†^ 95% CI	1.21 (1.06–1.37)	—	1.96 (1.69–2.27)	—	1.17 (1.02–1.34)	—	1.05 (0.84–1.31)	—	1.23 (1.08–1.39)	—	1.13 (1.01–1.26)	—	1.20 (1.05–1.37)	—
Poor mental health during the COVID–19 pandemic	% Yes, 95% CI	58.6 (52.7–64.5)	37.1 (33.7–40.4)	51.5 (47.8–55.3)	23.4 (19.9–26.8)	70.7 (65.9–75.6)	36.0 (32.7–39.4)	67.3 (55.8–78.8)	38.7 (35.3–42.1)	60.7 (54.7–66.7)	36.2 (33.2–39.3)	48.6 (43.5–53.6)	35.7 (32.5–38.8)	53.2 (48.9–57.6)	35.6 (32.3–38.8)
	aPR,* 95% CI	1.47 (1.32–1.63)	—	1.87 (1.65–2.12)	—	1.48 (1.34–1.63)	—	1.47 (1.25–1.72)	—	1.41 (1.29–1.54)	—	1.28 (1.17–1.41)	—	1.45 (1.33–1.57)	—
	aPR,^†^ 95% CI	1.11 (0.99–1.23)	—	1.68 (1.47–1.92)	—	1.19 (1.07–1.32)	—	1.07 (0.90–1.28)	—	1.15 (1.04–1.26)	—	1.17 (1.06–1.29)	—	1.20 (1.09–1.32)	—
Persistent feelings of sadness or hopelessness during the past year	% Yes, 95% CI	69.7 (64.3–75.2)	42.1 (38.8–45.4)	59.3 (55.5–63.0)	26.4 (23.3–29.4)	82.7 (78.1–87.3)	41.0 (37.7–44.3)	82.0 (72.9–91.2)	44.1 (40.7–47.6)	71.1 (67.2–75.0)	41.2 (37.7–44.7)	54.0 (49.1–58.8)	41.5 (38.1–44.9)	64.1 (59.7–68.5)	39.8 (36.2–43.3)
	aPR,* 95% CI	1.48 (1.36–1.60)	—	1.94 (1.75–2.14)	—	1.58 (1.46–1.70)	—	1.57 (1.41–1.76)	—	1.50 (1.37–1.63)	—	1.21 (1.11–1.32)	—	1.47 (1.33–1.62)	—
	aPR,^† ^95% CI	1.09 (0.99–1.19)	—	1.73 (1.56–1.93)	—	1.24 (1.14–1.35)	—	1.11 (0.97–1.27)	—	1.20 (1.10–1.31)	—	1.09 (1.01–1.18)	—	1.22 (1.10–1.35)	—
**Suicidal behavior**															
Seriously considered suicide during the past year	% Yes, 95% CI	42.2 (35.1–49.4)	18.3 (16.3–20.2)	30.7 (27.7–33.7)	7.9 (6.1–9.7)	53.6 (48.4–58.9)	17.3 (15.4–19.3)	56.9 (45.6–68.3)	19.9 (17.8–22.1)	41.7 (37.2–46.1)	17.8 (15.7–19.8)	25.6 (21.8–29.4)	19.0 (16.8–21.2)	33.7 (29.5–37.8)	17.3 (15.0–19.6)
	aPR,* 95% CI	1.89 (1.62–2.22)	—	3.13 (2.54–3.87)	—	2.22 (1.92–2.56)	—	2.21 (1.79–2.73)	—	1.81 (1.62–2.03)	—	1.23 (1.05–1.44)	—	1.71 (1.45–2.01)	—
	aPR,^† ^95% CI	1.20 (1.04–1.39)	—	2.61 (2.06–3.29)	—	1.55 (1.33–1.81)	—	1.25 (1.02–1.53)	—	1.25 (1.11–1.41)	—	1.06 (0.92–1.23)	—	1.26 (1.07–1.50)	—
Made a suicide plan during the past year	% Yes, 95% CI	34.6 (27.7–41.5)	13.6 (12.0–15.2)	24.0 (21.0–27.0)	5.2 (3.8–6.7)	46.0 (39.6–52.4)	12.6 (11.1–14.2)	46.4 (34.2–58.6)	15.1 (13.3–16.9)	35.4 (30.7–40.1)	12.9 (11.2–14.7)	19.5 (16.4–22.6)	14.5 (12.0–16.9)	26.6 (22.8–30.4)	12.9 (10.9–14.9)
	aPR,* 95% CI	2.04 (1.74–2.40)	—	3.62 (2.70–4.85)	—	2.59 (2.18–3.09)	—	2.34 (1.73–3.16)	—	2.14 (1.89–2.41)	—	1.20 (0.97–1.50)	—	1.74 (1.44–2.10)	—
	aPR,^† ^95% CI	1.24 (1.04–1.48)	—	2.89 (2.12–3.95)	—	1.75 (1.46–2.09)	—	1.18 (0.90–1.54)	—	1.41 (1.22–1.63)	—	1.02 (0.84–1.23)	—	1.24 (1.03–1.50)	—
Attempted suicide during the past year	% Yes, 95% CI	26.8 (21.7–32.0)	6.8 (5.5–8.1)	14.4 (12.0–16.7)	2.0 (1.3–2.7)	33.0 (27.0–39.0)	6.4 (5.3–7.5)	33.0 (22.3–43.6)	8.4 (6.9–9.8)	24.5 (20.2–28.9)	6.7 (5.5–7.9)	12.4 (9.1–15.6)	7.7 (6.3–9.0)	19.0 (15.9–22.1)	6.2 (4.9–7.5)
	aPR,* 95% CI	2.97 (2.45–3.60)	—	5.42 (3.81–7.71)	—	3.44 (2.59–4.58)	—	2.81 (1.82–4.35)	—	2.60 (2.11–3.21)	—	1.45 (1.04–2.00)	—	2.56 (2.03–3.22)	—
	aPR,^† ^95% CI	1.57 (1.29–1.91)	—	3.62 (2.55–5.14)	—	2.02 (1.50–2.73)	—	1.11 (0.70–1.75)	—	1.49 (1.20–1.83)	—	1.10 (0.82–1.48)	—	1.60 (1.23–2.08)	—

**Abbreviation:** aPR = adjusted prevalence ratio.

* Adjusted for sex, race and ethnicity, grade in school, sexual identity, and method of school instruction.

^† ^Adjusted for sex, race and ethnicity, grade in school, sexual identity, method of school instruction, and all other types of adverse childhood experiences.

## Discussion

Nearly three of every four U.S. high school students reported at least one ACE, and one in 13 (7.8%) reported four or more ACEs during the COVID-19 pandemic. Comparable prepandemic estimates of cumulative ACE exposure among U.S. adolescents are limited; estimates derived from prepandemic, retrospectively collected data among U.S. adults indicate that 60.9% reported at least one, and 15.6% reported four or more ACEs before age 18 years (*2*). Nevertheless, ACEs were common among U.S. adolescents during the pandemic and often resulted in acute consequences for mental health and suicidal behaviors, even among some adolescents who reported one to two ACEs. Consistent with literature indicating a dose-response relationship between ACEs and poor outcomes (*2*,*3*), after adjusting for demographic characteristics, the prevalences of poor current mental health and of past-year suicide attempts among adolescents who reported four or more ACEs during the COVID-19 pandemic were four and 25 times as high as those with no reported ACEs during the pandemic, respectively. The magnitude of effect associated with past-year suicide attempts is particularly concerning, given that a 2019 meta-analysis examining the association between having four or more ACEs and attempting suicide was considerably lower, albeit compelling (i.e., aOR = 7.3) (*3*). Differences in survey methods, ACEs assessed, exposure periods, and respondent age necessitate caution when comparing estimates. The findings of the current analysis are consistent with studies documenting the negative impacts of specific types of ACEs, including child abuse and neglect (*6*), sexual violence (*7*), teen dating violence (*7*), bullying (*7*), and family economic pressures (*8*). After considering demographic characteristics and ACEs assessed, emotional abuse was most strongly associated with poor mental health and suicidal behaviors. This finding is consistent with literature indicating that, although all forms of abuse contribute to adolescent suicidal behaviors, emotional abuse might be relatively more harmful (*9*).

General ABES limitations have been documented previously^††^ (*1*,*4*). The findings in this report are subject to five additional limitations. First, the cross-sectional nature of the data does not permit causal inferences in associations and might not account for all other factors affecting adolescent mental health or suicidal behaviors. Second, only seven ACEs were included in this analysis; inclusion of more ACEs^§§^ might have altered the ACE score distribution and which ACEs were most associated with mental health or suicidal behaviors. Third, this analysis did not directly assess experiences of or the impact of the pandemic, and instead included ACEs that adolescents commonly experience, including during the pandemic. Fourth, data on lifetime childhood exposure to ACEs were not available and might exacerbate or alter effects on mental health and suicidal behaviors for exposed children. Finally, the prevalences of some ACEs (e.g., teen dating violence), when comparable data are available, were lower in ABES^¶¶^ than 2019 Youth Risk Behavior Survey national estimates,*** possibly related to differences in methodology or behavior patterns during the COVID-19 pandemic. These differences could result in misclassification of ACEs exposure and have a potential impact on the strength of observed associations.

Concerns about poor adolescent mental health and suicidal behaviors preceded the COVID-19 pandemic (*10*) but escalated during the pandemic. For more than a decade, suicide has been the second or third leading cause of death among adolescents aged 14–18 years.^†††^ This analysis highlights the ongoing, urgent need to address adversity experienced before and during the pandemic to mitigate its impact on mental and behavioral health. CDC’s comprehensive approach to ACEs prevention and intervention emphasizes actions to create the structural and social environments that help children and families thrive, including bolstering family economic supports, supporting a strong start for children, and connecting youth and parents to community- and school-based resources^§§§^^,^^¶¶¶^ (*5*). Comprehensive, cross-sector approaches, partnerships, and policies focused on primary ACE****^,^^††††^ and suicide^§§§§^ prevention and intervention, including those focusing on early identification, linkage to care,^¶¶¶¶^ and access to trauma-informed services and supports, are necessary to improve adolescent mental and behavioral health.

**Summary**

**What is already known about this topic?**

Adverse childhood experiences (ACEs) are associated with poor mental health and suicidal behaviors.

**What is added by this report?**

The prevalences of poor current mental health and past-year suicide attempts among adolescents reporting four or more ACEs during the COVID-19 pandemic were four and 25 times as high as those without ACEs, respectively. Exposure to specific ACE types (e.g., emotional abuse) were associated with higher prevalences of poor mental health and suicidal behaviors.

**What are the implications for public health practice?**

Primary prevention and intervention strategies for ACEs and their acute and long-term impacts, including early identification and trauma-informed mental health service and support provision, could help address the U.S. child and adolescent mental health and suicide crisis.

## Footnotes

* ACEs are preventable, potentially traumatic events that occur in childhood (ages 0–17 years), such as neglect, experiencing or witnessing violence, and having a family member attempt or die by suicide. Also included are aspects of a child’s environment that can undermine their sense of safety, stability, and bonding. These examples do not comprise an exhaustive list of childhood adversity, as there are other traumatic experiences that can also impact health and well-being. https://www.cdc.gov/violenceprevention/aces/index.html and https://vetoviolence.cdc.gov/apps/aces-infographic/home.

† Multiple declarations related to the children and adolescents’ mental health and suicide crisis have occurred, including from federal authorities and national healthcare organizations. https://www.hhs.gov/sites/default/files/surgeon-general-youth-mental-health-advisory.pdf; https://www.aap.org/en/advocacy/child-and-adolescent-healthy-mental-development/aap-aacap-cha-declaration-of-a-national-emergency-in-child-and-adolescent-mental-health/

§
https://www.cdc.gov/healthyyouth/data/abes.htm

¶ Some items in ABES were asked with a past-year reference period. Because of the timing of the widespread onset of the COVID-19 pandemic (March 2020), and the period for ABES administration (January–June 2021), the past-year reference period generally reflects a similar, although not exact, time frame to questions that use the reference period “during the COVID-19 pandemic.”

** 45 C.F.R. part 46; 21 C.F.R. part 56.

††
https://www.cdc.gov/healthyyouth/data/abes/reports.htm

§§
https://www.cdc.gov/violenceprevention/aces/ace-brfss.html

¶¶ https://www.cdc.gov/healthyyouth/data/abes/tables/summary.htm#UIV (Accessed August 2, 2022).

*** https://nccd.cdc.gov/youthonline/App/Default.aspx (Accessed August 2, 2022).

††† https://www.cdc.gov/injury/wisqars/LeadingCauses.html (Accessed August 2, 2022).

§§§
https://vetoviolence.cdc.gov/apps/aces-training/#/

¶¶¶
https://www.cdc.gov/healthyyouth/whatworks/what-works-safe-and-supportive-environments.htm

****
https://www.cdc.gov/violenceprevention/aces/prevention.html

††††
https://www.cdc.gov/violenceprevention/childabuseandneglect/essentials/

§§§§
https://www.cdc.gov/suicide/pdf/suicideTechnicalPackage.pdf

¶¶¶¶
https://www.cdc.gov/childrensmentalhealth/documents/access-infographic.html

## References

[1] Jones SE, Ethier KA, Hertz M, Mental health, suicidality, and connectedness among high school students during the COVID-19 pandemic—Adolescent Behaviors and Experiences Survey, United States, January–June 2021. MMWR Suppl 2022;71(Suppl 3):16–21. 10.15585/mmwr.su7103a3 35358165 PMC8979602

[2] Merrick MT, Ford DC, Ports KA, Vital signs: estimated proportion of adult health problems attributable to adverse childhood experiences and implications for prevention—25 states, 2015–2017. MMWR Morb Mortal Wkly Rep 2019;68:999–1005. 10.15585/mmwr.mm6844e1 31697656 PMC6837472

[3] Petruccelli K, Davis J, Berman T. Adverse childhood experiences and associated health outcomes: a systematic review and meta-analysis. Child Abuse Negl 2019;97:104127. 10.1016/j.chiabu.2019.104127 31454589

[4] Krause KH, Verlenden JV, Szucs LE, Disruptions to school and home life among high school students during the COVID-19 pandemic—Adolescent Behaviors and Experiences Survey, United States, January–June 2021. MMWR Suppl 2022;71(Suppl 3):28–34. 10.15585/mmwr.su7103a5 35358164 PMC8979601

[5] CDC. Preventing adverse childhood experiences: leveraging the best available evidence. Atlanta, GA: US Department of Health and Human Services, CDC; 2019. https://www.cdc.gov/violenceprevention/pdf/preventingACES.pdf

[6] Angelakis I, Austin JL, Gooding P. Association of childhood maltreatment with suicide behaviors among young people: a systematic review and meta-analysis. JAMA Netw Open 2020;3:e2012563. 10.1001/jamanetworkopen.2020.12563 32756929 PMC7407092

[7] Baiden P, Mengo C, Small E. History of physical teen dating violence and its association with suicidal behaviors among adolescent high school students: results from the 2015 Youth Risk Behavior Survey. J Interpers Violence 2021;36:NP9526–47. 10.1177/0886260519860087 31271096

[8] Melchior M, Chastang JF, Falissard B, Food insecurity and children’s mental health: a prospective birth cohort study. PLoS One 2012;7:e52615. 10.1371/journal.pone.0052615 23300723 PMC3530436

[9] Miller AB, Esposito-Smythers C, Weismoore JT, Renshaw KD. The relation between child maltreatment and adolescent suicidal behavior: a systematic review and critical examination of the literature. Clin Child Fam Psychol Rev 2013;16:146–72. 10.1007/s10567-013-0131-5 23568617 PMC3724419

[10] Bitsko RH, Claussen AH, Lichstein J, Mental health surveillance among children—United States, 2013–2019. MMWR Suppl 2022;71(Suppl 2):1–42. 10.15585/mmwr.su7102a1 35202359 PMC8890771

